# Classification of Plank Techniques Using Wearable Sensors

**DOI:** 10.3390/s22124510

**Published:** 2022-06-14

**Authors:** Zong-Rong Chen, Wei-Chi Tsai, Shih-Feng Huang, Tzu-Yi Li, Chen-Yi Song

**Affiliations:** 1 Department of Athletic Performance, National University of Kaohsiung, Kaohsiung 811, Taiwan; english2320304@yahoo.com.tw; 2 Department of Education and Sport Sciences, College of Sports and Recreation, National Taiwan Normal University, Taipei 106, Taiwan; william-tsai@yahoo.com.tw; 3 Division of Physical Medicine and Rehabilitation, Zuoying Branch of Kaohsiung Armed Forces General Hospital, Kaohsiung 813, Taiwan; 4 Department of Applied Mathematics, National University of Kaohsiung, Kaohsiung 811, Taiwan; huangsf@nuk.edu.tw; 5 Institute of Statistics, National University of Kaohsiung, Kaohsiung 811, Taiwan; marycc812112@gmail.com; 6 Department of Long-Term Care, National Taipei University of Nursing and Health Sciences, Taipei 112, Taiwan

**Keywords:** core training, sports performance, kinematics, motion analysis, athlete, coaching

## Abstract

The plank is a common core-stability exercise. Developing a wearable inertial sensor system for distinguishing between acceptable and aberrant plank techniques and detecting specific deviations from acceptable plank techniques can enhance performance and prevent injury. The purpose of this study was to develop an inertial measurement unit (IMU)-based plank technique quantification system. Nineteen healthy volunteers (age: 20.5 ± 0.8 years, BMI: 22.9 ± 1.4 kg/m^2^) performed the standard plank technique and six deviations with five IMUs positioned on the occiput, cervical spine, thoracic spine, sacrum, and right radius to record movements. The random forest method was employed to perform the classification. The proposed binary tree classification model achieved an accuracy of more than 86%. The average sensitivities were higher than 90%, and the specificities were higher than 91%, except for one deviation (83%). These results suggest that the five IMU-based systems can classify the plank technique as acceptable or aberrant with good accuracy, high sensitivity, and acceptable specificity, which has significant implications in monitoring plank biomechanics and enabling coaching practice.

## 1. Introduction

Understanding sports biomechanics is important for injury prevention and performance enhancement, and wearable technology has the potential to positively influence coaching practice and athlete technique [1]. Evidence suggests that sensors are ideal for determining performance in sports during training or competition [2]. Wearable inertial sensor systems have been extensively validated to successfully measure the joint angle and temporal features during lower limb exercises [3]. Research over the past 10 years has predominantly focused on validating measurements that the systems produce, and on classifying the users’ exercise quality [3] for exercises such as squats and lunges [4,5], and for lower limb rehabilitation exercises such as knee extensions, straight-leg raises [6], and single-leg squats [7]. Wearable systems are also commonly used for monitoring and providing feedback on upper extremity movements during rehabilitation [8]. Additionally, wearable inertial sensor systems have been shown to provide a valid and reliable measure of postural control while performing the single- or double-leg static stance, tandem static stance, or balance test variations [9].

Core stability, the ability to control the position and motion of the trunk over the pelvis to allow optimum production, transfer, and control of force and motion to the terminal segment, plays a crucial role in athletic activities [10]. Core stability exercises have been commonly recommended in sports conditioning programs [11], as well as in rehabilitation, and are known to be effective in reducing pain intensity, functional disability, and improving quality of life, core muscle activation, and thickness in patients with non-specific chronic low back pain [12].

The plank (or prone bridge exercise), a traditional body-weight-bearing exercise, is a common core-stability exercise [13,14]. It provides adequate stimulus for endurance training of the rectus abdominis and external oblique abdominis [13]. The plank can also be used to evaluate overall core-muscle function [15,16]. Because the plank technique has many potential deviations [17], for example, the head is tilted forward or back at the neck, the pelvis is tilted back or dropped, the back is arched, or the hands are too close together, wearable inertial measurement units (IMUs) offer the potential for low-cost, objective biomechanical analyses that can be completed in a practical setting [1,2]. Applying objective quantitative or data-driven methods to detect aberrant movement patterns can enhance screening, assessment, and rehabilitation in sports, ergonomics, and medicine [18]. Furthermore, exercise training through wearables and Internet of Things (IoT) technologies are emerging. IMUs could allow the transfer of exercise data to a cloud-based server; the prescribed exercise technique and compliance could be assessed without the need for constant monitoring [5].

This study aimed to develop an IMU-based plank-technique quantification system. It was hypothesized that the system could distinguish between acceptable and aberrant plank techniques, as well as detect specific deviations from the acceptable plank technique.

## 2. Materials and Methods

### 2.1. Subjects

Nineteen healthy volunteers (age: 20.5 ± 0.8 years, height: 172.8 ± 5.8 cm, weight: 68.7 ± 6.3 kg, BMI: 22.9 ± 1.4 kg/m^2^) participated. The inclusion criteria included subjects (i) who could perform the plank technique for at least 30 s and (ii) had no lower and upper limb injuries in the past 6 months. Subjects with respiratory diseases during the experiment were excluded. All subjects were asked to maintain their lifestyle and refrain from alcohol, caffeine, and medicine 24 h prior to the experiment. This study was approved by the Human Research Ethics Committee of the Kaohsiung Armed Forces General Hospital. All participants provided informed consent after receiving a verbal explanation of the benefits, risks, and purposes of this study.

### 2.2. Procedures

**Figure 1:**
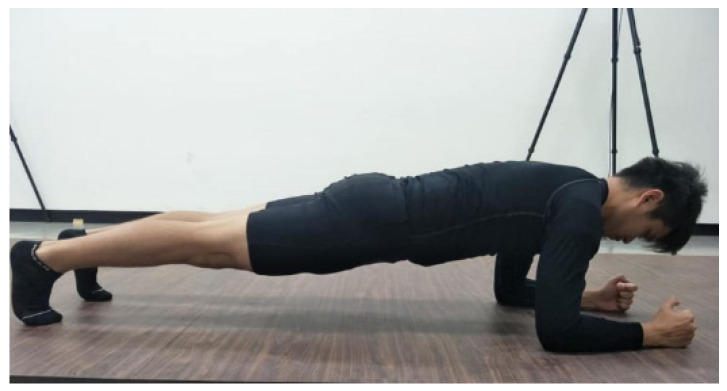
Acceptable plank technique. PC: The plank technique is acceptable.

**Figure 2:**
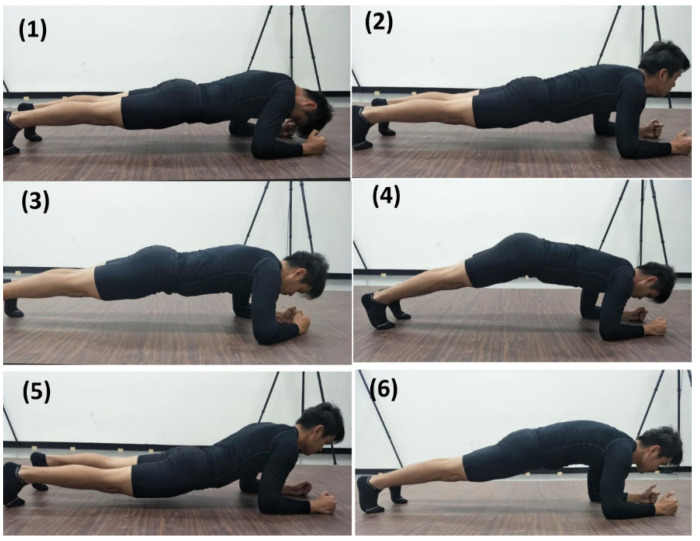
Six plank technique deviations. (**1**) HTFN: the head is tilted forward at the neck; (**2**) HTBN: the head is tilted back at the neck; (**3**) HC: the hands are too close; (**4**) HTB: the pelvis is tilted back; (**5**) HD: the hip is dropped; (**6**) BC: the back is convex.

Prior to the experiment, all subjects were asked to perform a dynamic warm-up comprised of 10 min of jogging at a self-selected pace and 10 min of dynamic stretching of major muscle groups. After completing the warm-up, the five IMUs (Xsens DOT, Enschede, The Netherlands), calibrated by the heading reset function, were placed in the same orientation and location at the occiput, C7, T3, sacrum, and midpoint of the right radius throughout the experiment. The subjects completed 10 s of the standard plank technique following the NSCA guidelines [19], that is, the body was supported on the palms, elbows, and toes with the spine in a neutral position while keeping the head, torso, and legs aligned (Figure 1). They were then instructed to complete six kinds of plank techniques with deviations, as outlined in the European Fitness Badge [17]. The test sequence is shown in Figure 2. Each aberrant plank technique was maintained for 10 s with a 30 s static sitting rest between them. An experienced athletic trainer confirmed that all plank techniques had been completed as instructed throughout the data collection.

### 2.3. Data Collection

**Figure 3:**
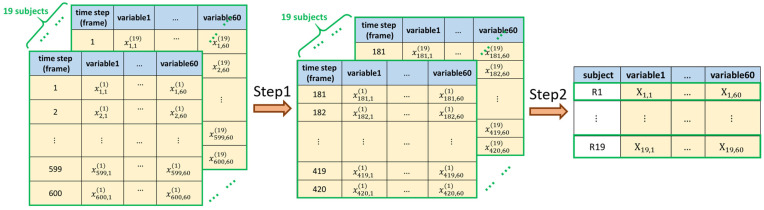
Diagram of the proposed data pre-processing.

Twelve variable sequences such as the Euler angle *x*, *y*, *z*, acceleration *x*, *y*, *z*, gyroscope *x*, *y*, *z*, and magnetometer *x*, *y*, *z* from each of the five inertia measurement units at timestamps 1,…, 600 (that is, 60 observations were collected each second for each variable) were collected for each plank technique. In other words, we collected a total of 60 variables, and each variable had 600 observations. Specifically, we used $x_{t,j}^{\left(k\right)}\;$ to denote the data of the jth variable, j=1,…,60 collected at time t, t=1,…,600 when the k-th subject, k=1,…,19 was performing each plank technique. To clarify this, we present the original data for each plank technique in the left panel of Figure 3, which indicates that the size of the original data for each plank technique was 600×60×19.

Because the objective was to distinguish plank techniques from the collected data, we conducted a suitable dimension reduction via data pre-processing, and designed an efficient and accurate classification model based on the reduced data. The details are as follows.

### 2.4. Data Analysis

Our data revealed that the pattern of $x_{t,j}^{\left(k\right)}$ behaved stably (or fluctuated less) during the period t∈(181,420) (i.e., the 4th to 7th second). Hence, Step 1 of our data pre-processing involved selecting the observations during this stable period to reduce the noise of the observations of the j-th variable for each subject, as shown in the middle panel of Figure 3. Next, to further reduce the dimension of the data, step 2 took the average of $x_{t,j}^{\left(k\right)},\;t\;=181,\ldots ,420$ to represent the j-th variable sequence for each subject, where the average is denoted by

(1) $$X_{k,j}=\frac{1}{240}\sum_{t=181}^{420}x_{t,j}^{\left(k\right)},$$

as shown in the right panel of Figure 3.

By implementing the above data pre-processing, we reduced the original data size from 600×60×19 to 19×60 for each type of plank technique, while maintaining the most helpful classification information for the next step of data processing.

We obtained two findings by investigating the distributions of the 60 variables for each plank technique. First, the plank technique HTBN can be perfectly separated from the others using the Euler-Y kinematic value collected from the first sensor (denoted by Euler-Y-S1). This finding inspired us to propose a binary tree-based classification model that sequentially separates the plank technique from the remaining techniques. Unfortunately, in addition to HTBN, the other six plank techniques cannot be separated perfectly by any single variable. Nonetheless, our second finding reveals that particular variables help filter out extremely aberrant performances for the other six plank techniques. Herein, we refer to these variables as filtered variables. In addition to filtering out extremely aberrant observations, we adopted the random forest algorithm, a nonlinear supervised learning method [20], to establish five binary classifiers using all 60 variables to separate the six plank techniques. In the following section, we introduce the details of arranging the orders of the plank techniques in the proposed binary tree model, the use of filtered variables to identify extremely aberrant observations, and the evaluation of the performance of the proposed method.

**Figure 4:**
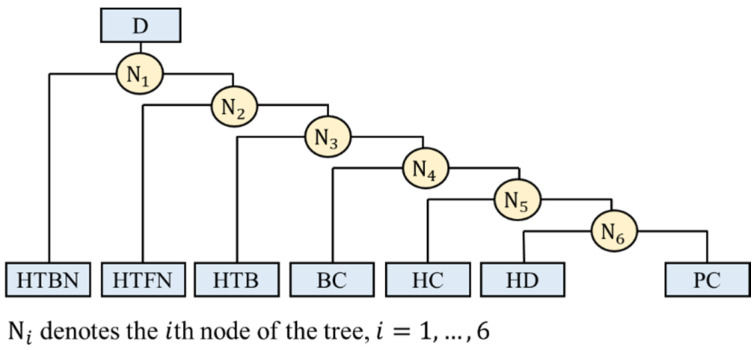
Proposed binary-tree classification model.

**Figure 5:**
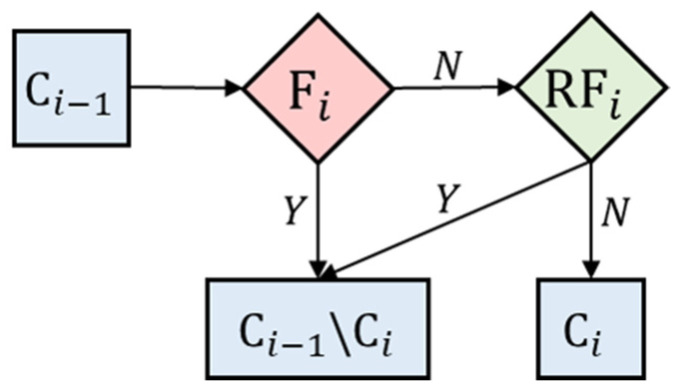
Flowchart of the two-stage procedure at $N_{i},$ i=2,…,6, where $C_{i}$ is defined in Table 1, $F_{i}$ denotes the stage of filtering extremely aberrant observations, and ${\mathrm{RF}}_{i}$ denotes the random forest classifier.

To simplify the illustration, we used Figure 4 to demonstrate the establishment of the proposed binary-tree-based classification model. According to our first finding, we separated the HTBN at the first node $N_{1}\;$ by the Euler-Y-S1 variable. If the unknown plank technique is classified as D\HTBN, where D is the set of all types of techniques and A\B denotes the removal of B from A, we continue to the 2nd node $N_{2}$. At $N_{2}$, we still had six different plank techniques to be separated, which indicated that there were six possibilities, and we needed to select one from them. The first stage of the proposed strategy, denoted by $F_{2}$, was used to identify extremely aberrant observations for each possibility using the corresponding filtered variables. The procedure for this step is described at the end of this section. Next, we built a random forest model, denoted by ${\mathrm{RF}}_{2}$, for each possibility, and selected the one with the highest classification accuracy to determine the plank technique that should be separated in $N_{2}$. The details of building the random forest model are presented in the next paragraph. The final result was to separate the HTFN from the other five types of plank techniques using $N_{2}$. By conducting a similar scheme at the 3rd, 4th, 5th, and 6th nodes, the proposed binary-tree-classification model is presented in Figure 4. Moreover, Figure 5 presents the flowchart of the above two-stage procedure at $N_{i}$, where $F_{i}$ denotes the stage of filtering out extremely aberrant observations, ${\mathrm{RF}}_{i}$ denotes the corresponding random forest classifier, and the dataset $C_{i}$ is defined in Table 1 below for i=2,…,6.

To introduce the scheme of building random forest classifiers at $N_{2},\ldots ,N_{6}$, we used the case of separating HTFN from the other five plank techniques at $N_{2}$ as an example for illustration. In this case, the desired random forest model aimed to separate two categories: HTFN (19 subjects) and the union of HTB, BC, HC, HD, and PC (95 observations). We randomly sampled approximately 80% of the subjects from each plank technique to form a training set, and the remaining subjects were treated as a test set. The training set was used to build a random forest model and the test set was used to evaluate the performance of the classifier. This 80-to-20% scheme is commonly adopted to evaluate classification performance in the fields of machine learning [21], social science [22], medical science [23], finance [24], and signal processing [25]. In the proposed procedure, because we randomly sampled approximately 80% of the data to learn random forest models from the 2nd to 6th nodes, different classification models may be obtained owing to different sampling results. Therefore, to objectively evaluate the classification performance of the proposed method, we independently repeated the proposed procedure 50 times. The results for the training and test sets are presented in Table 1 and Table 2, respectively.

In the following, we use the second node $N_{2}\;$ in Table 2 as an example to illustrate the computation of the classification accuracy, sensitivity, and specificity. In a test set at $N_{2}$, the HTFN category, denoted by $D_{1}$, has four subjects, and the other category, denoted by $D_{2}=D\backslash \mathrm{HTBN}$, has 20 observations (the other five types of plank techniques, each with four subjects). Let $n_{ij}$ denote the number of subjects classified as $D_{i}$ when their actual category is $D_{j}$, where i,j=1,2. Accordingly, the classification accuracy for the data was computed as $(n_{11}+n_{22})/\left(n_{11}+n_{12}+n_{21}+n_{22}\right)$, the sensitivity for correctly identifying HTFN was $n_{11}/\left(n_{11}+n_{21}\right)$, and the specificity was $n_{22}/\left(n_{12}+n_{22}\right)$. After independently performing this procedure 50 times, we present the average and standard deviation of the accuracies, sensitivities, and specificities separately for the 50 replications at $N_{2}$ in Table 2. A similar procedure can be used to obtain the average accuracies, sensitivities, and specificities, as well as the corresponding standard deviations at other nodes in Table 1 and Table 2.

Using the above notation, the extremely aberrant observations at each node are identified as follows. The core idea of this procedure is to compute the *p*-value of each filtered variable at each node. If the *p*-value of a filtered variable was <0.05, we classified the observation as an aberrant plank technique. The procedure for computing the *p*-value of the plank technique for any filtered variable is as follows:

1. For a fixed filtered variable in each replication, we used the values in set $D_{2}$ of the training data to establish an empirical distribution.
2. For each observation in the test set, we computed the *p*-value of the filtered variable based on the empirical distribution above.

For each $N_{2},\ldots ,N_{6}$ in Figure 4, we considered the original 60 variables collected from single sensors and some newly designed variables to capture the relationships across the sensors. Based on the above procedure, the filtered variables at each node are determined by selecting variables that are helpful in enhancing the classification performance of the entire system. The selected filtered variables are listed in Table 3.

## 3. Results

**Figure 6:**
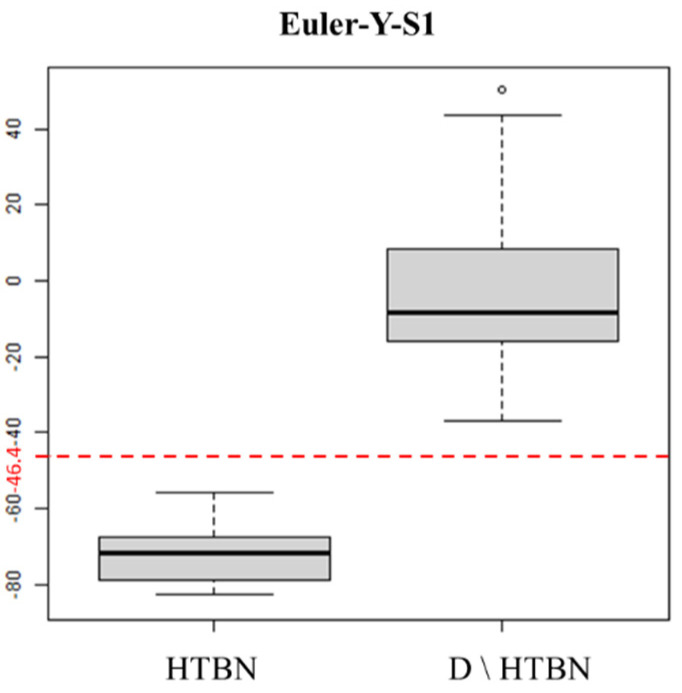
Boxplots of the Euler−Y kinematic values collected from the first sensor for HTBN and the other kinds of plank techniques (D\HTBN), where the values in the y−axis are computed from (1) and the circle denotes a potential outlier in the D\HTBN group.

Figure 6 presents boxplots of Euler-Y-S1 for HTBN and other plank techniques in D\HTBN at the $N_{1}$ node in Figure 4. The values of Euler-Y-S1 for HTBN were smaller than those of the other plank techniques. Hence, we can identify HTBN perfectly when observing a Euler-Y-S1 value less than −46.4, the average of the maximum in HTBN, and the minimum in D\HTBN.

Because the random forest already had satisfactory classification performance at $N_{2}$, Table 3 lists only the filtered variables at nodes $N_{3}$, $N_{4}$, $N_{5}$, and $N_{6}$, where one and two filtered variables defined by the original signals are adopted at $N_{3}$ and $N_{4}$, respectively. Particularly, four filtered variables were newly designed by taking the maximum of the Euler angles *z* measured by the 5th sensor (S5) and one of the other sensors at $N_{5}$. Similarly, six filtered variables are used at $N_{6}$ based on the Euler angle *z*, acceleration *x*, and magnetometer *z* measured by S3 and their relationships with other sensors. This phenomenon indicates that the relative relationships of the Euler angles *z* measured by S5 and the other sensors are helpful for identifying HC at $N_{5}$. Additionally, the Euler angle *z* and the relationships between the acceleration *x* and magnetometer *z* measured from the S3 relative to those measured from other sensors are helpful for identifying HD at $N_{6}$.

The accuracies at the six nodes were perfect for the training sets in 50 independent repetitions (Table 1). The proposed classification model can produce average accuracies above 86% with average sensitivities higher than 90% at all nodes for the test sets (Table 2). Specifically, five plank technique deviations, HTBN, HTFN, HTB, BC, and HC, were more easily identified than HD.

**Figure 7:**
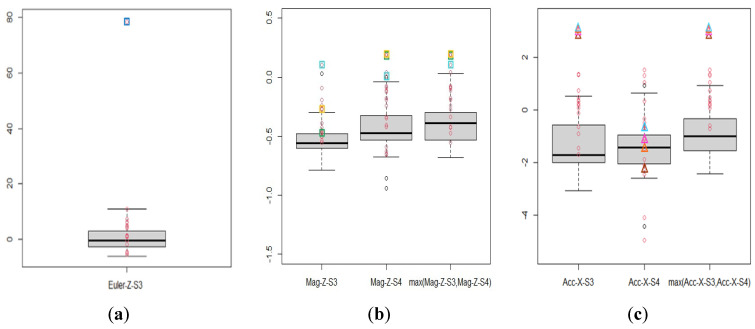
Boxplots of the filtered variables, (**a**) Euler−Z−S3, (**b**) max(Mag−Z−S3, Mag−Z−S4), and (**c**) max(Acc−X−S3, Acc−X−S4), for PC observations at $N_{6}$, where the values in the y−axis are computed from (1) using corresponding variables, the red circles denote the corresponding HD observations and those in color boxes or triangular are the detected extremely aberrant HD observations. In addition, the boxes (or triangular) in the same color are the observations of the same subject.

**Figure 8:**
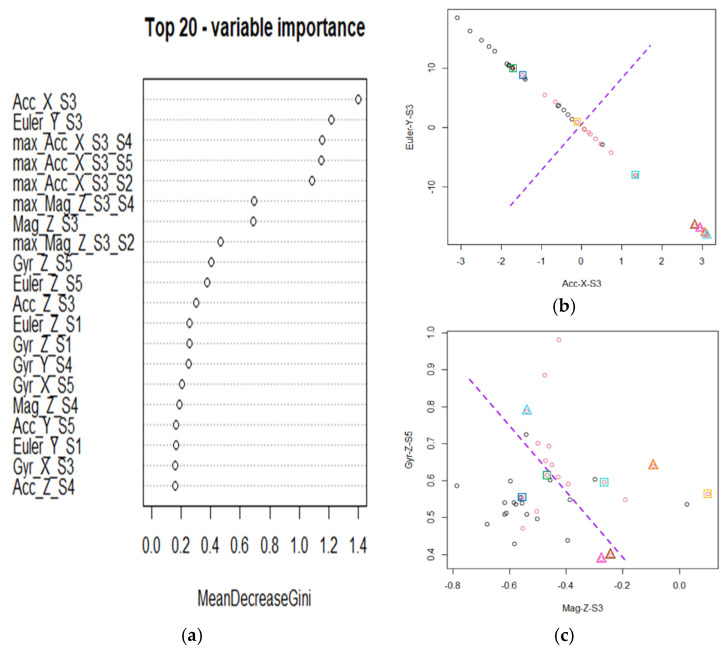
(**a**) The top 20 important classification variables of ${\mathrm{RF}}_{6}$, where the x-axis presents the mean decrease in Gini coefficient of each variable. (**b**) Classification diagram of the first two important variables of ${\mathrm{RF}}_{6}$, where the values in the x- and y-axis are computed from (1) using corresponding variables. (**c**) Classification diagram of the 3rd and 4th important variables excluding the 5 filtered variables designed from the original features of ${\mathrm{RF}}_{6}$, where the values in the x- and y-axis are computed from (1) using corresponding variables. In (**b**,**c**), the black (or red) circles represent the observations of PC (or HD).at $N_{6}$. The red circles in color boxes or triangular represent the extremely aberrant observations detected by the filtered variables listed in Table 3 for $N_{6}$. The dashed lines represent the classification boundaries. In addition, the boxes (or triangular) in the same color are the observations of the same subject.

Moreover, since the classification results of $N_{6}$ shown in Table 1 and Table 2 are the worst case, we employed the case at $N_{6}$ as an example for further discussion and presented the associated classification results in Figure 7 and Figure 8. Figure 7 presents the boxplots of the filtered variables, Euler-Z-S3, max(Mag-Z-S3, Mag-Z-S4), and max(Acc-X-S3, Acc-X-S4), for PC observations at $N_{6}$, where the red circles denote the corresponding HD observations. In addition, we adopted different-colored boxes or triangles to denote the extremely aberrant HD observations detected. One can see that 8 different extremely aberrant HD observations were detected by the 3 filtered variables. In particular, other filtered variables listed in Table 3 for $N_{6}$ detected the same extremely aberrant HD observations and had similar effects as shown in Figure 7b,c. Therefore, we employed max (Mag-Z-S3, Mag-Z-S4) and max (Acc-X-S3, Acc-X-S4) as examples to illustrate our findings for saving the space. In Figure 7b,c, the red circles in the two filtered variables, max(Mag-Z-S3, Mag-Z-S4) and max(Acc-X-S3, Acc-X-S4), especially for the latter one, are visually slightly more separable from their associated boxplots than the original four variables. Consequently, the two filtered variables can detect extremely aberrant observations and simultaneously have the potential to improve classification performances versus solely using original variables. This conjecture was confirmed in Figure 8a, which lists the top 20 important classification variables of ${\mathrm{RF}}_{6}$. Figure 8a reveals that the five filtered variables designed from the original variables for $N_{6}$ are also helpful classification variables with high ranks.

Furthermore, since ${\mathrm{RF}}_{6}$ is a nonlinear mapping of many features for separating PC and HD observations, it is not feasible to present the classification results of the proposed two-stage procedure via all classification variables. Therefore, we turn to present the classification diagrams of the important variables shown in Figure 8a according to the mean decrease in Gini coefficients, which measure the contribution of each variable to the homogeneity of the nodes and leaves in a random forest. The higher the value of the mean decrease Gini score, the higher the importance of the variable in the model. Specifically, Figure 8b presents the classification diagram of the first two important variables of ${\mathrm{RF}}_{6}$. In addition, excluding the five filtered variables designed from the original variables for ${\mathrm{RF}}_{6}$, Figure 8c further presents the classification diagram of the 3rd and 4th important variables of ${\mathrm{RF}}_{6}$. In Figure 8b,c, the black (or red) circles represent the observations of PC (or HD).at $N_{6}$. The red circles in color boxes or triangular represent the extremely aberrant HD observations detected by the filtered variables listed in Table 3 for $N_{6}$. The dashed lines represent the classification boundaries. One can see that around half of the detected extremely aberrant HD observations locate in complex zones for classification in Figure 8b or Figure 8c since they are mixed with black circles. This phenomenon explains why we proposed filtering out extremely aberrant observations before employing random forest for classification. After removing the eight extremely aberrant HD observations, Figure 8b,c indicate that ${\mathrm{RF}}_{6}$.can reasonably separate the two classes based on the perspective of the four important variables of ${\mathrm{RF}}_{6}$. Consequently, the proposed two-stage procedure was indeed helpful to enhance the classification sensitivity and accuracy at $N_{i}$, i=3,…,6.

## 4. Discussion

This study established a binary-tree-classification model to identify seven different plank techniques using the signals collected from five sensors placed on the occiput, C7, T3, sacrum, and midpoint of the right radius. The results demonstrated that the proposed model could identify six aberrant plank techniques with average sensitivities higher than 90% in the test sets. Additionally, the average specificities for the test data were greater than 91% for the first five nodes, except for the last one, which might be due to the small number of subjects at $N_{6}$, that is, $n_{1}=n_{2}=4$ in Table 2. Therefore, the current design of the placement of the five sensors and the proposed classification method can detect the six aberrant plank techniques with a high sensitivity and acceptable specificity.

To the best of our knowledge, this is the first study that has used inertial sensors to quantify the prone plank technique. The majority of previous research studied the ability of IMU-based systems to assess lower and upper limb exercises and balance activities [3,4,5,6,7,8,9], whereas a Korean study [26] utilized two inertial sensors attached to the thoracic spine and sacrum to quantify lumbar stability during wall plank-and-roll. With the previous finding of the in-field application of IMU-based systems, our results further support the use of plank technique assessment.

The correct execution of the prone plank technique involves maintaining the pelvis and spine in a neutral position while controlling the natural curvature of the spine [27]. In this study, our IMU-based plank technique quantification system achieved 97–100% accuracy, 91–100% sensitivity and 98–100% specificity in identifying neck deviations (i.e., HTBN and HTFN). This result is important because looking up with a hyperextended neck or the opposite, looking down, and throwing the cervical spine out of neutral alignment, stresses the cervical spine and strains neck muscles, leading to pain and injury. Moreover, the proper prone plank technique requires elbows to line up directly underneath the shoulder. Prone planks on forearms provide a larger base of support area upon which to distribute the weight compared to a high plank on hands. Incorrect alignment and positioning of the shoulder joints (e.g., HC) may alter the activation of the stabilizing muscles of the shoulder. Regarding common deviations in the shoulders, back, hips, and buttocks (e.g., HTB, HD, and BC), one study indicated that scapular positions (abduction or adduction) and pelvic positions (anterior or posterior tilt) modulated the core muscle activities (rectus abdominis, external oblique, internal oblique, and erector spinae) while performing the prone plank exercise [28], highlighting the greater contribution of these muscles to the postural stabilizing demands during posterior pelvic tilt positions, particularly when the scapulae are in adduction. Contrary to this challenging position, individuals in poor physical condition or those who perform a plank under fatigue might be at risk of shifting toward scapular abduction and the hyperkyphotic posture of the dorsal spine, a position that involves a lower demand of the core muscles [28]. Hence, our system has significant implications for monitoring the influence of changes in scapular and pelvic positions for better modulation and control of the intensity of the prone plank exercise.

The current study had several limitations. The testing sequence of the plank deviations was not randomized. Nevertheless, we provided participants with a between-testing rest period, and each plank technique was supervised by an experienced athletic trainer to minimize the possible contamination of the study results. Furthermore, the transferability from laboratory setting to realistic environment applications must be considered. The IMU systems presented may only produce these results when set up and analyzed in identical fashion to this study. While the dataset collected and analyzed in this study already included six common plank deviations, one in the real world may still present deviations that differ from the induced deviations evaluated in this study. Future studies may explore more plank forms, such as prone planks on the hands or knees and dynamic planks. With a larger sample size, future research could investigate gender effects to inform best practices for healthcare professionals. A health index may be calculated using the same plank as a metric by evaluating how long the subject manages to stay in the correct position.

## 5. Conclusions

The results of this study show that the five IMU-based systems can classify the plank technique as acceptable or aberrant with good accuracy, high sensitivity, and acceptable specificity, which has significant implications in monitoring plank biomechanics and enabling coaching practice.

## Figures and Tables

**Table 1:** Classification accuracies and corresponding standard deviations of the training sets at the six nodes in Figure 4.

Training Set				
$D_{1}$	$D_{2}$	$n_{1}$	$n_{2}\;$	Accuracy
HTBN	$C_{1}=D\backslash \mathrm{HTBN}$	15	90	100% (0.00)
HTFN	$C_{2}=C_{1}\backslash \mathrm{HTFN}$	15	75	100% (0.00)
HTB	$C_{3}=C_{2}\backslash \mathrm{HTB}$	15	60	100% (0.00)
BC	$C_{4}=C_{3}\backslash \mathrm{BC}$	15	45	100% (0.00)
HC	$C_{5}=C_{4}\backslash \mathrm{HC}$	15	30	100% (0.00)
HD	$C_{6}=C_{5}\backslash \mathrm{HD}=\mathrm{PC}$	15	15	100% (0.00)

Note: $D_{1}$ and $D_{2}$ denote the sets to be separated at each node, and $n_{1}$ and $n_{2}$ are the corresponding number of subjects in $D_{1}$ and $D_{2}$, respectively.

**Table 2:** Classification accuracies, sensitivities, specificities and the corresponding standard deviations of the testing sets at the six nodes in Figure 4.

Test Set						
$D_{1}$	$D_{2}$	$n_{1}$	$n_{2}\;$	Accuracy	Sensitivity	Specificity
HTBN	$C_{1}=D\backslash \mathrm{HTBN}$	4	24	100% (0.00)	100% (0.00)	100% (0.00)
HTFN	$C_{2}=C_{1}\backslash \mathrm{HTFN}$	4	20	97% (0.02)	91% (0.13)	98% (0.03)
HTB	$C_{3}=C_{2}\backslash \mathrm{HTB}$	4	16	93% (0.06)	92% (0.14)	93% (0.07)
BC	$C_{4}=C_{3}\backslash \mathrm{BC}$	4	12	91% (0.08)	92% (0.12)	91% (0.09)
HC	$C_{5}=C_{4}\backslash \mathrm{HC}$	4	8	93% (0.06)	94% (0.12)	93% (0.09)
HD	$C_{6}=C_{5}\backslash \mathrm{HD}=\mathrm{PC}$	4	4	86% (0.11)	90% (0.15)	83% (0.19)

Note: $D_{1}$ and $D_{2}$ denote the sets to be separated at each node, and $n_{1}$ and $n_{2}$ are the corresponding number of subjects in $D_{1}$ and $D_{2}$, respectively.

**Table 3:** Filtered variables for the nodes $N_{3}$, $N_{4}$, $N_{5}$, and $N_{6}$.

$N_{3}$	$N_{4}$	$N_{5}\;$	$N_{6}$
Gyr-Z-S3	Euler-Z-S3	max(Euler-Z-S5, Euler-Z-S1)	Euler-Z-S3
	Acc-X-S4	max(Euler-Z-S5, Euler-Z-S2)	max(Acc-X-S3, Acc-X-S2)
		max(Euler-Z-S5, Euler-Z-S3)	max(Acc-X-S3, Acc-X-S4)
		max(Euler-Z-S5, Euler-Z-S4)	max(Acc-X-S3, Acc-X-S5)
			max(Mag-Z-S3, Mag-Z-S2)
			max(Mag-Z-S3, Mag-Z-S4)

Note: We used the notation “Measurement-Direction-Sensor” to denote the four types of measurements (Euler angle, acceleration, gyroscope, and magnetometer), directions (X, Y, and Z), and sensors (S1, …, S5). Gyr = gyroscope, Acc = acceleration, Mag = magnetometer.

## Data Availability

The datasets used and/or analyzed during the current study are available from the corresponding authors on reasonable request.
